# Supplementary material for: COZOID: contact zone identifier for visual analysis of protein-protein interactions
Source: BMC Bioinformatics. 2018 Apr 6;19:125. doi: 10.1186/s12859-018-2113-6 (PMC5889581; doi:10.1186/s12859-018-2113-6)
Supplement: Supplementary file 2 — Software build. Executable binary file of the software tool. (ZIP 118,784 kb) [file 12859_2018_2113_MOESM2_ESM.zip › cozoid/java/docs/beansbinding-1.2.1-doc.zip!/org/jdesktop/beansbinding/class-use/Binding.SyncFailure.html]

Uses of Class org.jdesktop.beansbinding.Binding.SyncFailure (Beans Binding)


---


|  |  |  |
| --- | --- | --- |
| **Overview** \| **Package** \| **Class** \| **Use** \| **Tree** \| **Deprecated** \| **Index** \| **Help** | |  |
| PREV   NEXT | **FRAMES**    **NO FRAMES**     **All Classes** |  |


---


## **Uses of Class org.jdesktop.beansbinding.Binding.SyncFailure**

| Packages that use Binding.SyncFailure | |
| --- | --- |
| **org.jdesktop.beansbinding** | Provides support for defining properties and creating bindings between sets of two properties. |

Uses of Binding.SyncFailure in org.jdesktop.beansbinding

| Methods in org.jdesktop.beansbinding that return Binding.SyncFailure | |
| --- | --- |
| `Binding.SyncFailure` | `Binding.ValueResult.getFailure()`             Returns the failure if this `ValueResult` represents a failure and throws `UnsupportedOperationException` otherwise. |
| `Binding.SyncFailure` | `Binding.refresh()`             Fetches the value of the source property for the source object and sets it as the value of the target property for the target object. |
| `Binding.SyncFailure` | `Binding.refreshAndNotify()`             The same as `Binding.refresh()` with the additional behavior of notifying all registered `BindingListeners` with `synced` if `refresh` returns `null` or `syncFailed` if `refresh` returns a `SyncFailure`. |
| `protected  Binding.SyncFailure` | `Binding.refreshAndNotifyUnmanaged()`             A protected version of `Binding.refreshAndNotify()` that allows managed subclasses to refresh and notify without throwing an exception for being managed. |
| `protected  Binding.SyncFailure` | `Binding.refreshUnmanaged()`             A protected version of `Binding.refresh()` that allows managed subclasses to refresh without throwing an exception for being managed. |
| `Binding.SyncFailure` | `Binding.save()`             Fetches the value of the target property for the target object and sets it as the value of the source property for the source object. |
| `Binding.SyncFailure` | `Binding.saveAndNotify()`             The same as `Binding.save()` with the additional behavior of notifying all registered `BindingListeners` with `synced` if `save` returns `null` or `syncFailed` if `save` returns a `SyncFailure`. |
| `protected  Binding.SyncFailure` | `Binding.saveAndNotifyUnmanaged()`             A protected version of `Binding.saveAndNotify()` that allows managed subclasses to save and notify without throwing an exception for being managed. |
| `protected  Binding.SyncFailure` | `Binding.saveUnmanaged()`             A protected version of `Binding.save()` that allows managed subclasses to save without throwing an exception for being managed. |

| Methods in org.jdesktop.beansbinding with parameters of type Binding.SyncFailure | |
| --- | --- |
| `protected  void` | `Binding.notifySyncFailed(Binding.SyncFailure failure)`             Notifies all registered `BindingListeners` of a failure to sync (`refresh` or `save`), by calling `syncFailed` on each one. |
| `void` | `BindingListener.syncFailed(Binding binding, Binding.SyncFailure failure)`             Notification that the `Binding` attempted to sync the source and target, but the sync failed. |
| `void` | `AbstractBindingListener.syncFailed(Binding binding, Binding.SyncFailure failure)`             Notification that the `Binding` attempted to sync the source and target, but the sync failed. |

---


|  |  |  |
| --- | --- | --- |
| **Overview** \| **Package** \| **Class** \| **Use** \| **Tree** \| **Deprecated** \| **Index** \| **Help** | |  |
| PREV   NEXT | **FRAMES**    **NO FRAMES**     **All Classes** |  |


---
